# Nutritional status of iodine in pregnant women in Catalonia (Spain): study on hygiene-dietetic habits and iodine in urine

**DOI:** 10.1186/1471-2393-11-17

**Published:** 2011-03-08

**Authors:** Gemma Prieto, Maria Teresa Torres, Lidia Francés, Gemma Falguera, Lluis Vila, Josep María Manresa, Roser Casamitjana, Juan Ramón Barrada, Amèlia Acera, Dolors Guix, Anna Torrent, Josep Grau, Pere Torán

**Affiliations:** 1 Gerencia de Atención Primaria, Avenida Portugal 47, 05004 Ávila, Spain; 2 Atenció Salut Sexual i Reproductiva (ASSIR) Institut Català de la Salut Terrassa, CAP Antoni Creus i Querol, Carrer Italia 5, Terrassa, 08228 Barcelona, Spain; 3 Unidad Docente de Matronas de Cataluña, Escuela de Enfermería, Universitat de Barcelona, Feixa Llarga s/n, 08907 L'Hospitalet de Llobregat, Barcelona, Spain; 4 Atenció Salut Sexual i Reproductiva (ASSIR), Institut Català de la Salut Gerència Territorial Catalunya Central i Gerència Territorial Metropolitana Nord-Vallès Oriental- Valllès Occidental, Ctra de Barcelona 473, 1ª planta, 08203 Sabadell, Barcelona, Spain; 5 Servicio de Endocrinología y Nutrición, Hospital de Sant Joan Despí 'Moisès Broggi', Carrer Jacint Verdaguer 90, 08970 Sant Joan Despí, Barcelona, Spain; 6 Unitat de Suport a la Recerca Metropolitana Nord ICS-IDIAP Jordi Gol, Rambla 227, 08203 Sabadell, Barcelona, Spain; 7 Consultor Sènior del servei de Bioquímica i Genètica Molecular, Hospital Clínic, Carrer Villarroel 170, 08036 Barcelona, Spain; 8 Facultat de Psicología, Universitat Autònoma de Barcelona, 08193 Bellaterra, Barcelona, Spain; 9 Atenció Salut Sexual i reproductiva (ASSIR), Institut Català de la Salut Cerdanyola, Ctra. N-150 i C/Tarragona, s/n, 08291 Ripollet, Barcelona, Spain; 10 Atenció Salut Sexual i Reproductiva (ASSIR) Institut Català de la Salut Granollers, Moseu 19, 4ª planta, 08400 Granollers, Barcelona, Spain; 11 Atenció Salut Sexual i Reproductiva (ASSIR) Institut Català de la Salut Mollet, Av. Rívoli 7, 08100 Mollet, Barcelona, Spain; 12 Atenció Salut Sexual i Reproductiva (ASSIR) Institut Català de la Salut Osona, Pl Divina Pastora 6, 08500 Vic, Barcelona, Spain

## Abstract

**Background:**

It is a priority to achieve an adequate nutritional status of iodine during pregnancy since iodine deficiency in this population may have repercussions on the mother during both gestation and post partum as well as on the foetus, the neonate and the child at different ages. According to the WHO, iodine deficiency is the most frequent cause of mental retardation and irrreversible cerebral lesions around the world. However, few studies have been published on the nutritional status of iodine in the pregnant population within the Primary Care setting, a health care level which plays an essential role in the education and control of pregnant women. Therefore, **the aim of the present study **is: 1.- To know the hygiene-dietetic habits related to the intake of foods rich in iodine and smoking during pregnancy. 2.- To determine the prevalence of iodine deficiency and the factors associated with its appearance during pregnancy.

**Methods/design:**

We will perform a cluster randomised, controlled, multicentre trial. Randomisation unit: Primary Care Team. Study population: 898 pregnant women over the age of 17 years attending consultation to a midwife during the first trimester of pregnancy in the participating primary care centres. Outcome measures: consumption of iodine-rich foods and iodine deficiency. Points of assessment: each trimester of the gestation. Intervention: group education during the first trimester of gestation on healthy hygiene-dietetic habits and the importance of an adequate iodine nutritional status. Statistical analysis: descriptive analysis of all variables will be performed as well as multilevel logistic regression. All analyses will be done carried out on an intention to treat basis and will be fitted for potential confounding factors and variables of clinical importance.

**Discussion:**

Evidence of generalised iodine deficiency during pregnancy could lead to the promotion of interventions of prevention such as how to improve and intensify health care educational programmes for pregnant women.

**Trial Registration:**

ClinicalTrials.gov: NCT01301768

## Background

Iodine is a fundamental micronutrient for the organism which should be regularly administered through foods. Its function is essential for the synthesis of thyroid hormones which in turn act on the different organs and systems of the organism especially in the development of the central nervous system (CNS) from the earliest stages of embryonic and foetal development.

The ingestion of iodine depends on the type of foods consumed, their origin and preparation. Depending on the geographical area, products from the earth may have scarce iodine content. However, foods of marine origin are rich in this micronutrient [1]. In addition, it should be taken into account that foods lose iodine during their preparation: 20% is lost on frying, 23% on baking and 58% on boiling [2]. Some studies have shown that the iodine content of cows' milk differs according to what the animals are fed [3].

All these factors and alimentary habits make it difficult for the daily iodine requirements of the population to be covered through diet. Moreover, iodine is not stored in the body and must therefore be continually replenished. In normal conditions there is equilibrium between iodine intake and urinary elimination, and determination of iodine in urine (ioduria) constitutes a good indicator of iodine intake [4], with assessment of ioduria in a casual urine sample providing adequate information on the nutritional status of iodine [5].

During pregnancy there is an increase in thyroid hormone requirements due to the physiological modifications produced in response to the metabolic demands of pregnancy. This increase can only be achieved by a proportional increase in hormone production which directly depends on the availability of iodine in the diet. Moreover, gestation produces a physiological increase in the elimination of iodine in the urine because of a rise in glomerular filtration. In cases with an underlying deficit in iodine these modifications of pregnancy may not be compensated leading to failure of the mechanisms of adaptation. It is therefore very important to increase iodine intake from the beginning of gestation and even beforehand if possible, similar to the recommendations of supplementation of folic acid. The thyroid hormones available for embryonic and foetal tissue during the first trimester of gestation depend exclusively on maternal hormones and thus, a deficit in maternal iodine can have negative repercussions on prenatal development [6].

According to the World Health Organization (WHO) together with the United Nations International Children's Emergency Fund (UNICEF) and the International Council for Control of Iodine Deficiency Disorders (ICCIDD) the iodine needs of pregnant women have been established as 200 μg/L/day [4]. The ICCIDD has recently raised this recommendation to 250-300 μg/L/day. Maternal iodine intake may be calculated by the determination of ioduria (level of iodine in urine) taking into account that the factor of dilution in urine is greater in pregnant women than in the remaining population. The value of ioduria indicating optimum iodine intake during gestation should be between 150 and 230 μg/L [7]. Studies carried out in different European countries such as France, England, Germany, Switzerland, Ireland and Hungary have demonstrated the highly variable values of iodine deficiency (values less than 150 μg/L) in pregnant women, from 3.5% in England to 57.1% in Hungary [8-13].

Another aspect to consider within hygienic-dietetic habits of pregnant women is smoking and the repercussion this may have on maternal thyroid function. Smoking is considered a goitrogenic substance since it inhibits the absorption of iodine during both gestation and the period of lactation [14]. Smoking during gestation is associated with changes in the levels of thyroid function in both the mother and the foetus. The concentration of Thyroid-stimulating Hormone (TSH) in the mother (in the first and third trimester of gestation) and in the blood of the umbilical cord are lower and the T3 levels are higher which may trigger adverse effects for both [15,16]. It has also been reported that smoking during the period of lactation increases the risk of iodine deficiency which may lead to brain damage in the lactating child [14].

Iodine deficiency is not only a problem in developing countries but also affects most of the industrialised countries to a greater or lesser degree. It has currently been estimated that half of the population lives in areas in which there is a risk of having disorders due to iodine deficiency such as what occurs in several European countries such as Germany, Belgium, Denmark, Spain, France, Greece, Ireland and Italy. Zones with endemic goitre or some other alterations related to iodine deficiency have been detected in Spain [17-19].

Until several years ago the fundamental problem of iodine deficiency lay in endemic goitre but in the last decades studies have demonstrated a wide spectrum of disorders caused by iodine deficiency during pregnancy such as an increase in the number of abortions and dead foetuses, an increase in neonatal morbimortality and hearing defects in infants [20], a reduction in intellectual capacity and growth, congenital abnormalities with permanent neuromotor damage [21,22] as well as the attention deficit syndrome and hyperactivity [23,24]. According to the WHO, lack of iodine is the most frequent cause of mental retardation and irreversible brain lesions in the world [4].

Recent studies have demonstrated that iodine intake during gestation is low, even in areas where theoretically there are institutional prevention programmes to promote the consumption of iodised salt [18,25-27]. All these studies have been determining factors for the Spanish and the Catalan Societies of Endocrinology and Nutrition, the Spanish Society of Gynaecology and Obstetrics and the Ministry of Health and Social Policies to consider pregnant women as a risk group for disorders as a consequence of iodine deficit and to thereby recommend a diet rich in iodine and iodised supplements. Iodine deficiency in this population, albeit moderate, represents a high risk of cerebral lesions in the foetus [19,22].

The contribution of the quantity of iodine necessary for a good health status has been included as a basic human right: "All children have the right to an adequate intake of iodine to ensure normal development", especially children yet to be born and thus, "All mothers have the right to adequate iodine ingestion during gestation to ensure that their child achieves an optimum mental development" [28,29].

Some of the interventions most frequently studied during gestation have been the cessation of smoking and the promotion of maternal breastfeeding. McBride [30] hypothesised that pregnancy may be an "appropriate time for learning" to give up smoking since there is a greater perception of risk in this period and of personal results in pregnancy which provoke affective or strong emotional response and redefines social function or the self-conception of a woman, especially when failure in fulfilment of a social function results in social stigmatisation. This "appropriate time for learning" is not only appropriate for smoking cessation but also for any other health habit of the pregnant woman such as, for example, alimentary habits.

Some studies have demonstrated that the effectiveness of individual versus group Education for Health is similar [31-33] in different populational groups (diabetics, infant asthmatics or pregnant women), while others have reported favourable results in group interventions [34,35].

The interest in group education has grown in the last years, although very few studies have formally evaluated their effectiveness compared with individual education. In general, group versus individual education carries a reduction in health care costs in hospitalisations, medical consultations and the consumption of drugs for some chronic diseases such as diabetes mellitus and rheumatic diseases as well as different aspects of maternal-foetal health [36].

However, there are numerous difficulties involved in the development of group education such as for example the inadequate or imprecise definition of the educational objectives, the difficulties in evaluating the process and the results as well as the variable attendance of the patients and the limitations in the material and human resources available. Nonetheless, there is sufficient evidence to clearly indicate that these interventions are possible and effective.

The relevance of this study lays not only in knowing the type of educational intervention (group or individual), with modification of alimentary habits in pregnant women being the most effective, but also in performing a follow up of both hygienic-dietetic habits in the consumption of iodine rich foods and ioduria during the three trimesters of gestation. It is particularly of note that in the first trimester, the critical time in embryonic development, midwives in primary care centres should carry out a health care education intervention (individual or group) on these habits with special emphasis on the consumption of foods with high iodine content and iodine supplements.

If the results obtained by this study demonstrate the presence of generalised iodine deficiency during gestation, new studies would be required in other areas of Catalonia and the rest of Spain and would support the need to implement systematic programmes of iodine supplementation during gestation as well as the incorporation of specific educational programmes on alimentary habits.

## Hypothesis

The hygienic-dietetic habits of a pregnant woman has repercussion on both the nutritional status of the mother and the development and growth of the foetus. According to data obtained in a previous study carried out within the Primary Care setting of Catalonia, there is reduced consumption of foods rich in iodine since 71% report eating fish less than three times per week, 18% do not drink milk daily, only 42% use iodised salt for food condimentation and preparation and 18% of the pregnant women surveyed continue to smoke during gestation. We therefore consider that:

### 1st HYPOTHESIS

Most pregnant women in Catalonia have hygienic-dietetic habits which involve reduced intake of foods containing iodine (such as the eating of fish and the use of iodised salt for food condimentation) and in addition, an important percentage of these women continue to smoke during gestation.

### 2nd HYPOTHESIS

Most of the pregnant women in Catalonia have ioduria (levels of iodine in urine) with values of less than 150 μg/L (value considered as iodine deficiency) taking into account that the elimination of iodine in urine during gestation rises due to a physiological increase in glomerular filtration.

## Objectives

### General objective 1

o know the hygieninc-dietetic habits with respect to the consumption of iodine-rich foods and smoking of women during the three trimesters of gestation.

### General objective 2

To know the prevalence of ioduria less than 150 μg/L (iodine deficiency) and the factors associated with its appearance during the first three months of gestation.

### Secondary Objectives

1. Validate the questionnaire on nutritional iodine status in pregnant women.

2. Determine the iodine-rich foods consumed by pregnant women in each trimester of gestation and how these foods are prepared (boiled, fried, baked...).

3. Determine whether pregnant women use iodised salt in the preparation and condimentation of foods.

4. Determine whether pregnant women take any supplement containing iodine during gestation.

5. Describe the modifications in the smoking habits produced during gestation.

6. Compare the nutritional iodine status of the pregnant population between the different Primary Care Centres (PCC) participating in the study, ethnic groups, levels of education and gestational trimester.

7. Study the repercussions of the educational interventions on hygienic-dietetic habits and smoking as well as on iodurias and the taking of iodine supplements.

## Methods/Design

### Design

Cluster randomised, controlled, multicentre trial. Randomisation unit: primary care team (PCT).

### Setting

46 PCTs from the province of Barcelona that provide health coverage to urban, semirural and rural populations.

### Study population

All the pregnant women over the age of 17 years attending consultation to a midwife during the first trimester of gestation in the different participating PCC during the study period (2007-2010).

### Exclusion criteria

Pregnant women in the second or third trimester of gestation; pregnant women with diagnosed thyroid disease; pregnant women without a telephone; pregnant women with communication difficulties (cognitive or sensory deterioration, language barrier); pregnant women who do not consent to participate in the study.

### Sample size calculation

The sample size has been calcuated by multipying the size of a randomized simple design by the design effect. For the simple randomized design an alpha error of 0.05 and a beta error of 0.20 in a bilateral contrast are considered, requiring 204 pregnant women in the intervention group and 204 in the non intervention group to detect a difference greater than or equal to 15% in the prevalence of iodine deficiency between the two. After extensive review of the literature on group educational interventions in a pregnant population, the investigative team [37-40] considered that the intervention to be undertaken in the intervention group will achieve an improvement of at least 15% in hygienic-dietetic habits and thus in the levels of ioduria. A proportion of iodine deficiency of 44% in the non intervention group is assumed as indicated by studies performed in populations in the Pyrenees and the coast of the Maresme, areas near the study setting [18]. A loss to follow up of 15% has been estimated. Using an intracluster correlation coefficient of 0.05 [41-43], and based on an average of 25 pregnant women per PCT, the design effect is 2.2. Therefore, 898 pregnant women and 36 PCT are needed.

### Data Collection

*Dependent Variables*: iodine deficiency (yes, no), is defined as ioduria less than 150 μg/lL

Independent Variables:

Variables related to the woman (information obtained through a individualized interview):

- Administrative: PCT, midwife, data collection date, patient code, telephone number.

- Sociodemographic: date of birth, level of education, etnia.

- Obstetric: n° of pregnancies brought to term; n° premature births, n° of miscarriges and n° of live births, trimester of gestation of the woman (1st, 2nd or 3rd).

- Related to smoking: smoker (no, yes), cigarettes/day prior to gestation, cigarettes/day during gestation, ex-smoker because of pregnancy, ex-smoker and time since cessation of smoking.

- Related to consumption of iodine-rich foods:

▪ Consumption of milk: glasses/day

▪ Consumption of yoghourt: units/week and type (whole or skimmed).

▪ Consumption of cheese: rations/week and type (cured, semi-cured and fresh).

▪ Consumption of vegetables: times/week, usual preparation (raw, fried, grilled/baked, steamed, boiled) and use of water in which they have been boiled (yes, no and no answer).

▪ Consumption of vegetables and garden produce: times/week.

▪ Consumption of fish: times/week, usual preparation (raw, fried, grilled/baked, steamed, boiled).

▪ Consumption of tinned tuna: times/week.

▪ Consumption of tinned sardines: times/week.

▪ Consumption of meat and derivatives: times/week.

▪ Consumption of eggs: units/week.

▪ Consumption of fruit: units/week and types (strawberries, pineapple, peaches, bananas, oranges and others).

▪ Consumption of nuts: times/week.

- Consumption of iodised salt: yes (time of consumption), no.

- Consumption of multiple vitamins containing iodine: yes (type), no.

- Consumption of potassium iodine supplement: yes (type), no.

- Related to the intervention: study group (intervention, non intervention), attendance to group workshop (yes, no).

Variables related to the midwife (information obtained through a individualised interview): years in the profession, primary care nursing training, mean number of visits per day.

Variables related to the PCT (information obtained through an individualised interview): population assigned, mean age of population assigned, whether or not it is a teaching center for residents, number of midwives.

### Description of the Study

The phases of the study are:

* **PHASE 1. Recruitment of primary care nurses (PCN)**: the project was presented to all the PCN within the study setting to recruit midwives interested in participating. Those who were interested in collaborating signed a document of commitment to participate in the study.

* **PHASE 2. Creation of the study groups (intervention-IG and non intervention-NIG) and midwife training programme**:

- The randomisation unit was the PCN. Using simple randomisation the centres distributed the IG and NIG at a proportion of 1:1.

- The formation of the participating midwives was carried out on 3 centralised work days during which the develpment of the study was explained in depth and they were given instructions on correct completion of the questionnaire on hygienic-dietetic habits in both a paper and electronic format. Each professional was assigned a support investigator to clarify any doubts which may arise during the study period. The objective of session was for the midwives to obtain sufficient training to avoid possible variability in the development of the study and in the collection and registration of data.

* **PHASE 3. Pilot study**: this was carried out in three randomly selected PCN with 30 pregnant women who were not included in the study in order not to affect the final results. This pilot study evaluated the study inclusion process of the pregnant women, data collection by paper and electronic questionnaire as well as the appointment circuit, referral and presentation of ioduria results.

* **PHASE 4. Recruitment of the pregnant women, initial evaluation and request for first ioduria (in IG and NIG)**:

- Recruitment of the pregnant women: the pregnant women will be consecutively recruited from the women attending consultation to the midwife until the number of 25 women required by each PCN has been reached. Each midwife will know the number of women that they must recruit for the study. If a pregnant woman fulfils the inclusion criteria the midwife will explain the study to her and will ask for her participation. If the woman accepts she will be given the study information sheet in which the objectives and the characteristics are described and she will be asked to sign the informed consent form to thereby be included in the study. If the woman refuses to participate the reasons for this will be noted, as will the administrative, sociodemographic and obstetric variables with the aim of determining the profile of this type of pregnant woman.

- Initial evaluation of the pregnant women: on the first contact with the women and through an individualised interview the midwife will collect the following variables: administrative, sociodemographic, obstetric, those related to smoking, consumption of foods rich in iodine, iodised salt and iodised multiple vitamins. All the data will be reported in an electronic data collection file (EDCF) which will include the rule of internal coherence to guarantee the quality control of the data. Following the interview the midwife will make an educational intervention on the importance of good alimentation during pregnancy and the repercussions this has on maternal and foetal health, with special emphasis on the importance of the consumption of foods rich in iodine and iodised supplements. An informative sheet will also be provided on the importance of iodine during gestation.

- Request for ioduria during the first trimester: the midwife will request an ioduria test and will explain the woman how to correctly collect the urine and will program the appointment for the second trimester visit of the pregnancy. On receipt of the ioduria results the midwife will note these in the EDCF. In cases in which iodine deficiency is detected the midwife will duly intervene, thus the reason for including the telephone number of the pregnant woman in the questionnaire. If iodine supplementation is indicated the administration will begin on collection of the first urine sample to avoid underestimation of the prevalence of iodine deficiency in the first trimester. Ioduria will be analysed using the Benotti&Benotti method performed in the laboratory of the Hospital Clinic of Barcelona.

* **PHASE 5. Group Education Intervention (only in IG)**: the coordinating midwives in each zone will be responsible for performing the educational workshops on hygienic-dietetic habits in the pregnant women in the intervention group. This workshop will be held in the first trimester since this is when organogenesis is produced and is therefore the time of greatest risk for the appearance of alterations in the development of the foetal central nervous system in cases of maternal iodine deficiency. The content of the educational workshop will be the same to avoid a variation in information and transmission of knowledge to the pregnant women, that is, to ensure receipt of homogeneous information by the pregnant women.

* **PHASE 6. Follow up (in IG and NIG)**: if a pregnant woman does not attend the appointment she will be called by telephone and if she cannot be contacted after 10 attempts she will be withdrawn from the study. The interview on hygienic-dietetic habits will be given in the second and third trimester appointments and determination fo ioduria will be requested. The reasons for participant withdrawal will be reported and a flow chart of the patients will be created throughout the study.

* **PHASE 7. Validation of the questionnaire on the iodine nutritional status in pregnant women**: the questionnaire has been elaborated by the investigator team after extensive review of the literature and finally based on the alimentary table according to iodine content (mcg/100 g) included in the monography on "Los alimentos y la salud" (Food and Health) (Egura R. Webb S. Tovar JL. Y Gausí C. Los minerales y la salud. Barcelona: Nuevas Ediciones de Bolsillo, S.L.; 2000. p 161-97). The questionnaire describes the foods with the greatest iodine content and the quantities consumed, the use of iodised salt and multiple vitamins containing iodine and smoking since the latter is a goitrogenic substance. Neither the origin of the foods nor the place in which the food is prepared is taken into account because of the impossibility to evaluate and confirm these aspects, despite the influence in the quantity of iodine consumed. Validation will be undertaken by an expert in psychometry from the Autonomous University of Barcelona (UAB) taking into account ioduria values.

* **PHASE 8. Completion of data collection**: after performing the third trimester appointment data collection will be concluded. The periodic controls of the data reported in the EDCF will have localised and corrected any incoherences that may have arisen.

* **PHASE 9. Data analysis and diffusion of the results**: the data obtained will be analysed and a final report will be elaborated with diffusion of the results in different scientific scenarios.

### Statistical analysis

Data will be analysed in concordance with the Consort Cluster guide [44], and all analyses will be done on an intention to treat basis. A descriptive analysis and an analysis of baseline comparability between the study groups will be performed for the variables studied. A Student's t test or a Mann-Whitney U test will be used to compare means with two categories. An ANOVA test will be done to compare means with two or more categories, and a Chi- squared test or a Chi -squared tendency test will be done to compare categorical variables. A multilevel logistic regression will be carried out to evaluate the association between the dependent and the independent variables that were statistically significant in the bivariate analysis. All analyses will be adjusted for potential confounding factors and for variables with clinical relevance. All tests will be done with a 95% bilateral confidence interval. SPSS 15, STATA 10 and HLM6 will be used.

### Quality Control

Several procedures are employed to ensure the quality of the study data, thus maximizing the validity and reliability of the program delivery and outcome assessments. These are:

• Formation of the professionals participating in the study.

• Use of an electronic data collection system with a system of warnings and internal coherency norms.

• Regular meetings and mailings between members of the study group and all participating centres.

### Ethical Aspects

In the first contact with the pregnant woman, she will be given information about the study and will sign an informed consent form if she wishes to participate. The informed consent will describe the ethical conditions and the participant's right to intimacy, anonymity, confidentiality, withdrawal and information. The investigators are committed to respecting the norms of good clinical practice, as well as the requirements of the Helsinki Declaration. The protocol has been evaluated and approved by the Ethical Committee of Scientific Research of the Primary Care Research Institute Jordi Gol (Barcelona, Spain). Confidentiality of data: Only the investigators and monitors/auditors of the study will have access to the data of the subjects who agreed to participate.

## Discussion

One of the strengths of the present study is its design which is pragmatically oriented with respect to the needs of time and material resources.

Another strength of the study is the large number of midwives and patients involved, reinforcing the external validity of the data.

Since no register of women in the first trimester of pregnancy is available, selection of the study population can not be approached with a simple randomised sample but will rather be performed by consecutive sampling which could lead to a selection bias, the self-selection of the volunteer. To determine the possible presence of such a bias the administrative, sociodemographic and obstetric variables of the women who refuse to participate in the study will be analysed.

To increase the validity and reliability of the data collection of the hygienic-dietetic habits by individualised interview, the midwives have been especially trained in this task following a training session prior to initiation of the pilot study and a support investigator will be available to solve any doubts which may arise.

The difficulty in determining iodine intake through diet will be compensated by the determination of iodine levels in urine which is considered a good marker of iodine ingestion according to the WHO, UNICEF and ICCIDD [4,45].

To avoid the phenomenon of contamination which may appear when the intervention influences the subjects in the control group (frequent when the same work centre of the professionals belongs to different study groups), randomisation of the intervention will be performed by the PCN and not by study subjects. Although the unit of randomisation is the primary care unit, the intervention is given at the individual level and the outcomes are also measured at this level. The estimation of the design effect will be taken into account in all the main analyses.

Since participant losses and withdrawals randomly occur, any subject exclusion from the analysis may later affect group comparability. To preserve this all the patients included in the study will be evaluated according to the intention to treat principle and thus, all the patients will be analysed as pertaining to the group to which they were initially assigned independently of the treatment they have actually received.

Lastly, it should be pointed out that if the questionnaire on the nutritional status of iodine is found to be valid and reliable, it may be a valuable tool for the detection of iodine deficiency during gestation in midwife consulting offices without the need to request ioduria. The midwife may thereby intervene rapidly and adequately in such a situation informing the woman as to the role of iodine in foetal development and underline the importance of foods rich in iodine.

If the results obtained demonstrate the presence of iodine deficiency in an important proportion of the gestating population, this would support the implementation of preventive interventions to improve and intensify health care education programmes in pregnant women as well as to make the public health authorities aware of the importance and repercussions of iodine deficiency and attempt to systematically implement supplementation of foods containing iodine, a measure which already prevails in some countries such as Argentina.

## Abbreviations

**(CNS)**: Central Nervous System; **(EDCF)**: Electronic Data Collection File; **(ICCIDD)**: International Council for Control of Iodine Deficiency Disorders; **(IG)**: Intervention Group; **(NIG)**: Non Intervention Group; **(PCC)**: Primary Care Centres; **(PCN)**: Primary care nurses; **(PCT)**: Primary Care Team; **(TSH)**: Thyroid-stimulating Hormone; **(UNICEF)**: United Nations International Children's Emergency Fund; **(WHO)**: World Health Organization;

## Competing interests

he authors declare that they have no competing interests

## Authors' contributions

GP, MTT, LF, GF and LV formulated the study question. GP, MTT, LF, GF, LV, JMM, JRB participed in the revision of the bibliography and design of the study methodology. MTT, GF, JMM. RC and PT colaborated carrying out the study. GP, MTT, LF, LV, JMM and PT contributed in the writing and preparation of the present manuscript. All of the authors have read and approved of the present manuscript. The members of the Iodegest Group are participating in recruitment and follow-up of the patients.

## Author Details

The IODEGEST Study Group:

**PASSIR Sabadell**: (Coordination Montse Abella). Nuria Sampedro, Glòria Miralpeix, Montse Villanueva, Concepción Manzano, Judit Cos.

**PASSIR Cerdanyola**: (Coordination Pilar Soteras). Fermina Casas, Coloma Graells, Mireia Llucià, Rosalia Ibars.

**PASSIR Mollet**: (Coordination Encarna López). Montserrat Manzanares, Irene Lorente, Eva Artieda, Meritxell Casajoana, Dolors Muñoz, Llucia Burgos, Angelica Hidalgo.

**PASSIR Osona**: (Coordination Anna Fusté). Dolors Lladó, Rosa Subirats, Angelina Masoliver, Imma Trujillo, Rosa Banús, Dolors Salas, Montse Pujol, Dolors Grau, Roser Sanglas, Anabel Mayos.

**PASSIR Bages**: (Coordination Náyade Crespo). Rosa Codina, Rosa Forn, Montserrat Galí, Antonia Hidalgo, Teresa Macià, Mercè Vendrell, Montserrat Sallent, Montserrat Ribera, Rosa Oller, Teresa Riba, Esther Romero, Adelaida Expósito, Encarnació Santaeulàlia, Anna Vilaseca.

**PASSIR Granollers**: (Coordination Dolors Guix). Gemma Olivera, Merche García, Rosa Sans, Marta Roman, Mª Jose Vila, Maite Martinez, Esther Serrano, Sonia Díaz, Carolina Alcaine, Susana Sancho, Remei Fenollosa, Encarna Gascón, Núria Risques, Araceli Santamaria, Remei Corominas, Xavi Espada, Maria Helena Perez, Concepción de la Fuente, Assumpta Prats, Maria Rosa Cabedo, Carme Magem, Mercedes Vigil, Carmen Biern, Montse Bach, Joana Relat.

**PASSIR Mútua Terrassa**: (Coordination Engracia Coll). Carmen Bayascas, Olga Ezquerro, Patricia Reategui, Anna Campos, Rosa Bach.

**PASSIR Anoia**: (Coordination Inés Molina). Eva Martinez, Anna Bartolí, Rosa Ferrer, Rocio Hernandez.

## Pre-publication history

The pre-publication history for this paper can be accessed here:

http://www.biomedcentral.com/1471-2393/11/17/prepub
